# Time Course of Left Ventricular Strain Assessment via Cardiovascular Magnetic Resonance Myocardial Feature Tracking in Takotsubo Syndrome

**DOI:** 10.3390/jcm13113238

**Published:** 2024-05-30

**Authors:** Hiroki Goto, Ken Kato, Yoichi Imori, Masaki Wakita, Noriko Eguchi, Hiroyuki Takaoka, Tsutomu Murakami, Yuji Nagatomo, Toshiaki Isogai, Yuya Mitsuhashi, Mike Saji, Satoshi Yamashita, Yuichiro Maekawa, Hiroki Mochizuki, Yoshimitsu Takaoka, Masafumi Ono, Tetsuo Yamaguchi, Yoshio Kobayashi, Kuniya Asai, Wataru Shimizu, Tsutomu Yoshikawa

**Affiliations:** 1 Department of Cardiovascular Medicine, Chiba University Graduate School of Medicine, 1-8-1 Inohana, Chuo-ku, Chiba 260-8677, Japan; moritap77@gmail.com (H.G.);; 2 Department of Cardiovascular Medicine, Nippon Medical School Hospital, Tokyo 113-8603, Japan; s9012@nms.ac.jp (Y.I.);; 3 Department of Cardiovascular Medicine, Tokai University School of Medicine, Isehara 259-1193, Japan; 4 Department of Cardiology, National Defense Medical College, Tokorozawa 359-8513, Japan; 5 Department of Cardiology, Tokyo Metropolitan Tama Medical Center, Tokyo 183-8524, Japan; 6 Department of Cardiology, Sakakibara Heart Institute, Tokyo 183-0003, Japan; 7 Department of Cardiovascular Medicine, Toho University Graduate School of Medicine, Tokyo 143-8541, Japan; 8 Division of Cardiology, Internal Medicine III, Hamamatsu University School of Medicine, Hamamatsu 431-3192, Japan; 9 Department of Cardiovascular Medicine, St. Luke’s International Hospital, Tokyo 104-8560, Japan; 10 Department of Cardiovascular Center, Toranomon Hospital, Tokyo 105-8470, Japan

**Keywords:** takotsubo syndrome, takotsubo cardiomyopathy, cardiovascular magnetic resonance, feature tracking, strain

## Abstract

**Background:** Although takotsubo syndrome (TTS) is characterized by transient systolic dysfunction of the left ventricle (LV), the time course and mechanism of LV function recovery remain elusive. The aim of this study is to evaluate cardiac functional recovery in TTS via serial cardiac magnetic resonance feature tracking (CMR-FT). **Methods:** In this Japanese multicenter registry, patients with newly diagnosed TTS were prospectively enrolled. In patients who underwent serial cardiovascular magnetic resonance (CMR) imaging at 1 month and 1 year after the onset, CMR-FT was performed to determine the global circumferential strain (GCS), global radial strain (GRS) and global longitudinal strain (GLS). We compared LV ejection fraction, GCS, GRS and GLS at 1 month and 1 year after the onset of TTS. **Results:** Eighteen patients underwent CMR imaging in one month and one year after the onset in the present study. LV ejection fraction had already normalized at 1 month after the onset, with no significant difference between 1 month and 1 year (55.8 ± 9.2% vs. 58.9 ± 7.3%, *p* = 0.09). CMR-FT demonstrated significant improvement in GCS from 1 month to 1 year (−16.7 ± 3.4% vs. −18.5 ± 3.2%, *p* < 0.01), while there was no significant difference in GRS and GLS between 1 month and year (GRS: 59.6 ± 24.2% vs. 59.4 ± 17.3%, *p* = 0.95, GLS: −12.8 ± 5.9% vs. −13.8 ± 4.9%, *p* = 0.42). **Conclusions:** Serial CMR-FT analysis revealed delayed improvement of GCS compared to GRS and GLS despite of rapid recovery of LV ejection fraction. CMR-FT can detect subtle impairment of LV systolic function during the recovery process in patients with TTS.

## 1. Introduction

Takotsubo syndrome (TTS) is a clinical syndrome characterized by an acute and transient left ventricular (LV) systolic dysfunction often related to an emotional or physical stressful event [1]. LV dysfunction and wall motion abnormality in the acute phase represent apical ballooning and wall motion abnormalities that extend beyond the territory perfused by a single coronary artery and the absence of culprit coronary artery disease lesions [2]. TTS has been recognized as a disease where contraction abnormalities are completely reversible and accompanied by recovery of LV function [3]. However, some studies suggested that the relatively rapid recovery of LV ejection fraction (LVEF) after an acute attack of TTS was not paralleled by symptomatic recovery [4], and echocardiographic indices, for example, global longitudinal strain (GLS) and apical circumferential strain, remained abnormal after a few months [5].

Cardiovascular magnetic resonance (CMR) imaging is helpful for the evaluation of the comprehensive assessment of the functional and structural changes that occur in TTS patients [6]. CMR feature tracking (CMR-FT) was introduced as a new method to quantify regional LV deformation similar to speckle-tracking echocardiography [7]. CMR-FT is useful for evaluating LV deformation of TTS, and the transient contraction abnormalities in TTS can be quantitatively assessed with CMR-FT [8]. Some studies reported residual alterations of cardiac deformation in TTS patients using two-dimensional strain echocardiography [9]. A few data have been published on residual changes in cardiac function of patients with prior recovered TTS using CMR-FT showing deformation abnormalities that may persist despite normalization of global LV function at follow-up of 3 months [8]. However, no prior studies have comprehensively evaluated the recovery process of left ventricular systolic function during long-term follow-up within 1 year in TTS using CMR-FT. In our study, we evaluated cardiac functional recovery of post-acute TTS between a month and a year using CMR-FT.

## 2. Methods

### 2.1. Patients

In the current study, we included patients with newly diagnosed TTS who visited one of 8 study centers between September 2017 and January 2021. To be eligible for enrollment, patients had to meet the following criteria: first, the presence of a transient abnormality in LV wall motion beyond a single epicardial coronary artery perfusion territory; second, the absence of obstructive coronary artery disease or evidence of acute plaque rupture in coronary angiography or coronary computed tomography; third, the presence of new electrocardiographic abnormalities or elevation in cardiac troponin levels; fourth, the absence of myocarditis or pheochromocytoma [10,11]. An exception to these criteria is the focal (within one coronary distribution) type and death during the acute phase before wall motion recovery in a patient matching all other criteria. If coronary artery disease is found, the diagnosis of TTS can still be made if the wall motion abnormalities are not in the distribution of the coronary disease. 

Investigators at each institution obtained approval from the local institutional review board before participating in the study. Each patient was fully informed about the study and provided written consent to participate. In the case of adults lacking sufficient decision-making capacity, an explanation was provided to their families.

### 2.2. Study Design

This prospective cohort study was conducted at 8 centers in Japan for TTS and the study design conformed to the ethical principles of the Declaration of Helsinki. Patients were prospectively enrolled using a data-capture system in each institution and anonymized data were collected through the coordinating office and the data center. The registry was registered under the University Hospital Medical Information Network Clinical Trials Registry (UMIN000029363), and the first member of the writing committee assumed responsibility for the accuracy and completeness of the data and for the fidelity of the study to the protocol. 

### 2.3. Data Collection

All patients with TTS who were hospitalized at a study center during the enrollment period and met these criteria were invited to join the study. Local investigators prospectively identified eligible patients, and those who agreed to participate were enrolled. The investigation was conducted by physicians at each facility who entered data into a database. The database was designed to collect baseline data and follow-up data obtained through clinical and laboratory evaluations performed as part of routine clinical practice. The patient’s clinical characteristics, symptoms at the onset and triggers of TTS were recorded at admission by the accepting physician. Blood tests and electrocardiogram (ECG) are checked on admission. Ballooning patterns were identified via echocardiography and/or left ventriculography and classified as apical ballooning (typical), mid-ventricular, basal or focal type. Transthoracic echocardiography (TTE) was performed in all patients during hospitalization. In-hospital outcomes were checked when discharged. Outpatient visits were conducted after discharge was performed one month and one year after the onset. Clinical follow-up data were obtained during outpatient visits. Long-term outcome data were collected from clinical visits one year after the onset. Serial CMR imaging was performed around 1 month and 1 year after the onset. 

### 2.4. Cardiac Magnetic Resonance Acquisition 

Standardized imaging was conducted using 1.5 or 3.0 Tesla magnetic resonance scanners (in Nippon Medical School Hospital: Achieva, Philips, the Netherlands; in Tokyo Metropolitan Tama Medical Center: MAGNETOM Sola, Siemens Healthineers, Germany; in Chiba University: Ingenia 3.0T Omega HP, Philips, The Netherlands; in Hamamatsu University: Discovery MR750, GE Healthcare Technologies, Chicago, IL, USA). Our standardized protocol included cine images (steady state-free precession sequences). An LV 4-chamber view, a 2-chamber view and a stack of short-axis views in each series were obtained during beath-holds gated to the ECG. LV function, including LVEF, LV end-diastole volume (LVEDV), LV end-systole volume (LVESV), LV mass and LV stroke volume (LVSV), were evaluated by tracing endocardial and epicardial borders in biplane cine CMR images during the cardiac cycle. The myocardial mass was evaluated during the diastole phase. 

### 2.5. Cardiovascular Magnetic Resonance Myocardial Feature Tracking

CMR-FT was performed by a doctor (H.G.) experienced in this field at Chiba University using dedicated evaluation software (MR Wall Motion Tracking Multi Chamber V8.7, Canon Medical Systems, Japan). CMR offline analyses were carried out for evaluation of LV deformation at one month and one year after the onset. Endocardial and epicardial borders were manually drawn at end-diastole frames in short- and long-axis sequences using a point-and-click approach. After that, the border tracking algorithm was automatically applied. The algorithm tracked image features throughout the cardiac cycle. Accurate tracking was verified via visual review, and manual adjustments or reapplication of the algorithm were performed, if necessary. Peak longitudinal strain was based on long-axis two- and four-chamber view analyses. Short-axis views at basal, midventricular and apical locations were analyzed to derive peak circumferential strain and peak radial strain. Global CS (GCS) and global RS (GRS) were calculated from the mean values from segmental evaluations. GLS was averaged strains quantified from two- and four-chamber views. 

### 2.6. Statistical Analysis

All statistical analyses were performed using EZR version 1.52 (Saitama Medical Center, Jichi Medical University, Saitama, Japan) [12], which is a graphical user interface for R (The R Foundation for Statistical Computing, Vienna, Austria). Continuous data were expressed as mean ± standard deviation or median (interquartile range). Categorical data were expressed as frequency with percentage. A paired sample *t*-test was used to evaluate changes in LV strain values one month and one year after the onset in the same patient. A value of two-sided *p* < 0.05 was considered statistically significant.

## 3. Results

### 3.1. Main Patients’ Characteristics

Ninety-five patients diagnosed with TTS were initially enrolled in this study from 8 hospitals. Baseline characteristics and clinical events are summarized in Tables S1 and S2. Finally, we analyzed the CMR images of 18 patients from 4 centers who underwent CMR imaging one month and one year after the onset (Table 1). Regarding the 18 TTS patients, 14 patients (77.8%) were female and the average age was 75.1 ± 10.4 years. The most common morphological type of TTS was an apical ballooning type (61.1%). The most common trigger of TTS was physical stress. Most patients experienced symptoms, with 83.3% presenting with typical symptoms such as chest pain or dyspnea. A past history of psychological disease was reported in 2 patients (11.1%) and a history of neurologic disease in 1 patient (5.6%). The average LVEF on admission assessed via echocardiography or left ventriculography was 46.5%, and 8 patients (44.4%) showed reduced LVEF (40% or less). Regarding oral medications, the proportion of patients taking beta-blockers (50.0% vs. 16.7%) and angiotensin-converting-enzyme inhibitor or angiotensin-receptor blocker (44.4% vs. 22.2%) was higher at discharge compared to admission. Clinical events in hospitalization are summarized in Table 2. Heart failure was observed in 8 patients (44.4%). Of these patients, 3 patients required ventilator support. Cardiogenic shock developed in 3 patients (16.7%). All patients with cardiogenic shock required catecholamine and an intra-aortic balloon pump was used in 2 patients (11.1%). No patients had left ventricular thrombus in our cohort. One patient (5.6%) experienced complete atrioventricular block, while ventricular tachycardia was not observed in any patients. 

### 3.2. Cardiac Magnetic Resonance

**Figure 1:**
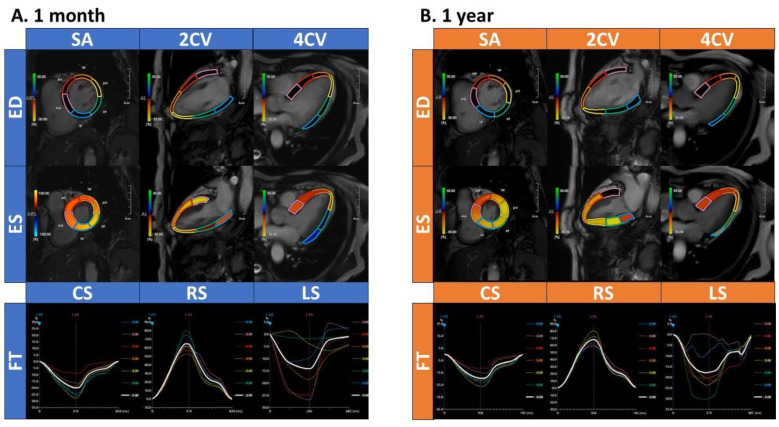
Analysis of circumferential, radial and longitudinal strain via cardiac magnetic resonance feature tracking.

**Figure 2:**
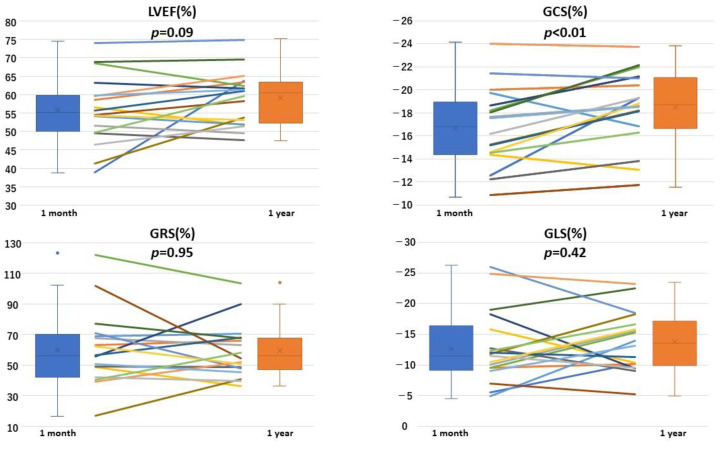
Comparison of ejection fraction and global circumferential strain, global radial strain and global longitudinal strain between 1 month and 1 year after the onset of takotsubo syndrome. The cross symbol represents the mean value. The dot symbol represents outliers.

The first CMR imaging was performed 10–83 days (median 36.5 days) after the onset and the second CMR imaging was performed 273–373 days (median 358.5 days) after the onset. CMR findings are summarized in Table 3. LVEF had already normalized at one month after the onset, with no significant difference between one month and one year (55.8 ± 9.2% vs. 58.9 ± 7.3%, *p* = 0.09). LVEDV, LVESV, LV mass and LVSV showed no significant differences between one month and one year (LVEDV: 81.5 ± 27.0 mL vs. 82.2 ± 22.7 mL, *p* = 0.91; LVESV: 36.4 ± 15.2 mL vs. 34.2 ± 12.9 mL, *p* = 0.54; LV mass: 45.1 ± 15.5 g vs. 48.0 ± 12.8 g, *p* = 0.41; LVSV: 84.4 ± 27.1 mL vs. 80.9 ± 25.1 mL, *p* = 0.33). Figure 1 shows a representative case who underwent CMR-FT analysis. CMR-FT demonstrated that there was a significant improvement in GCS from one month to one year (−16.7 ± 3.4% vs. −18.5 ± 3.2%, *p* < 0.01), while GRS and GLS had already normalized at one month and showed no significant difference between one month and one year (GRS: 59.6 ± 24.2% vs. 59.4 ± 17.3%, *p* = 0.95, GLS: −12.8 ± 5.9% vs. −13.8 ± 4.9%, *p* = 0.42) (Figure 2). 

CMR-FT in short axis views quantifies CS and RS, and in long axis views quantifies LS in patients who experienced TTS. Figure 1A,B show CMR-FT analysis at 1 month and 1 year after the onset of TTS, respectively. The analysis divided the myocardium in each slice into six segments and evaluated the strains in each segment. In each coordinate system, curves are color-coded to correspond to each segment, with the colors reflecting the analyzed strain values. The white lines represented the average of CS, RS and LS in each segment in the slice. CMR-FT = cardiac magnetic resonance feature tracking; CS = circumferential strain; RS = radial strain; LS = longitudinal strain; TTS; takotsubo syndrome; ED = end-diastole; ES = end-systole; CV = chamber view; SA = short axis; FT = feature tracking.

The average GCS significantly increased between one month and one year after the onset, while the average LVEF, GRS and GLS did not show a significant increase during the same period. LVEF = left ventricular ejection fraction; GCS = global circumferential strain; GRS = global radial strain; GLS = global longitudinal strain. 

## 4. Discussion

In the present study, we have shown that GCS significantly improved from one month to one year, as demonstrated via CMR-FT. Conversely, LVEF, GRS and GLS exhibited no significant change from one month to one year after the onset. 

TTS was first described in 1990 by Sato et al. [13]. The exact pathophysiological mechanisms of TTS are incompletely understood, despite the initial report being introduced about 30 years ago. However, there are some proposed mechanisms underlying TTS, such as sympathetic stimulation, multi-vessel epicardial spasm and microvascular dysfunction [3,14]. Sympathetic stimulation is considered as central to its pathogenesis because an emotional or physical factor is associated with the onset of TTS [11]. 

The diagnosis of TTS is often difficult because TTS has various variants, and its clinical presentation may closely resemble acute myocardial infarction regarding ECG abnormalities and biomarkers [10]. Various diagnostic criteria for TTS have been established, including the Mayo Clinic Diagnostic Criteria and the Inter TAK Diagnostic Criteria [3,10]. LV dysfunction plays a key role in the diagnosis of TTS. Patients with TTS showed various morphological patterns and can primarily be classified into four major types (apical, midventricular, basal and focal) based on the distribution of regional wall motion abnormalities [11,15]. All types of TTS exhibit spontaneously reversible LV systolic dysfunction [13,16]. Therefore, these criteria all include evidence of transient LV dysfunction (hypokinesia, akinesia or dyskinesia). LV dysfunction recovers to apparently normal wall motion within weeks in most cases [14,17]. Similarly, the LVEF also improves correspondingly. In a recent cohort studied, the average LVEF improved from 41.1% to 59.9% within 2 months [11]. However, recent studies have reported incomplete LVEF recovery from TTS, characterized by residual systemic inflammation and increased cardiac mortality at follow-up [18]. The recovery time for TTS patients is also clinically important. TTS patients with delayed LV recovery had less favorable outcomes compared to patients with early recovery [19]. These patients exhibited an increased rate of ventilation, ventricular thrombus formation and cardiogenic shock compared to patients with early recovery. Recovery time for TTS patients depends on various factors. Previous studies demonstrated that delayed LV recovery was associated with different characteristics [19]. The reasons for delayed LV recovery included male sex, reduced LVEF, acute neurologic events and the manifestation of TTS due to physical stress. Global left ventricular myocardial strain in TTS also recovered, similar to LVEF [8]. In the present study, we demonstrated that GCS significantly improved from one month to one year, as demonstrated via CMR-FT. However, we could not evaluate risk factors for delayed recovery of GCS because of the small sample size of the current study. Further studies with a larger number of patients are needed to focus on the time course of recovery of strain parameters in more detail and detect risk factors for delayed recovery. 

Additionally, despite the recovery of LVEF and wall motion, persistence of microscopic changes at the cellular level and diastolic dysfunction were indicated [4,20]. T2-weighted signal intensity on CMR imaging suggesting myocardial edema and inflammation in TTS also persists even after the improvement of wall motion abnormalities [21]. Recent studies have reported the persistence of symptoms and elevated brain-type natriuretic peptide release well beyond this period, even after the improvement of LV dysfunction in TTS [22,23], suggesting an association with these residual cardiac dysfunctions. These findings highlight that in addition to the improvement of cardiac wall motion abnormality and LVEF, evaluating other aspects of cardiac function is also crucial for understanding the patient’s condition after TTS. In addition to the assessment of LVEF and wall motion abnormalities, a comprehensive evaluation, including strain on TTE and CMR imaging, is considered important. This is because complete myocardial recovery is a slower process compared to the normalization of the LVEF and resolution wall motion abnormalities [24]. 

Several reports have indicated the effectiveness of strain analysis for LV dysfunction in TTS using CMR-FT [8,25]. Speckle tracking echocardiography (STE) has also been considered an accurate method to assess myocardial strains. Many studies demonstrated that, concerning myocardial strain parameters based on CMR-FT and STE, there was good agreement between these modalities [26,27,28,29]. GLS appeared to be more accurate with STE, while GCS showed a better reproducibility with CMR-FT [25]. In patients with TTS during the acute phase, GLS, GCS and GRS exhibit significant differences compared to the healthy control group, as well as previously reported normal values in healthy individuals [8,30,31]. At 3.5 months after the onset, regarding the apical ballooning type of TTS, all global strain values showed significant improvement [8]. GLS and GCS reached levels considered to be within the normal range, whereas GRS remained below the cutoff [8,30,31]. In the present study, we focused on the time course of changes in LV strain in TTS patients with recovered LVEF and no wall motion abnormalities using CMR imaging performed at 1 month and 1 year after the onset. In our analysis, GCS significantly improved between one month and one year after the onset, while GLS and GRS did not significantly change during this period. This result indicated that circumferential myocardial motion may improve more slowly than longitudinal and radial myocardial motion. In accordance with our results, a previous study demonstrated that GLS evaluated via TTE in patients with TTS recovered to the normal range throughout the 5 weeks after the onset [32]. These results suggest that the peak improvement in GLS occurs around one month after the onset. While the mechanism underlying the delayed recovery of GCS compared to GLS and GRS in TTS remains unclear, similar observations have been reported in the recovery process of sepsis-induced cardiomyopathy and post-surgical aortic valve replacement in aortic stenosis, suggesting a potential role of myocardial structural composition [33,34]. Additionally, there has been a report indicating the effectiveness of GCS assessment using echocardiography in predicting prognosis for patients with dilated cardiomyopathy, suggesting a potential association between delayed GCS recovery and prognosis in TTS as well [35]. TTS patients with impaired strain parameters in spite of recovered LVEF might need closer monitoring compared to those with normal strain parameters. The extent of strain changes after normalized wall motion abnormalities in patients with TTS remains poorly reported, and further study is necessary. 

### Study Limitations

The present study has some limitations. We did not perform a comparison of the LV strain values between TTS patients and a population of healthy individuals. The values of LV strain evaluating CMR imaging are influenced by various factors including the analysis software. Few reports exist on the normal LV values in Japanese individuals as analyzed via the software used in this study. This is because the comparison of LV strain values between our data and the previously reported normal ranges is challenging. However, it is believed that a relative assessment of LV strain changes within the same individual is possible, and thus, this study is considered to be insightful. Our data include various types of TTS including apical balloon, midventricular, basal and focal types. The time courses of LV strains may differ among TTS types. Furthermore, due to the limited number of cases in this study, evaluation of outcomes could not be conducted. To further investigate the differences in recovery processes between different morphological types and the association between CMR-FT indices and prognosis, and establish personalized treatment strategies, larger-scale studies are warranted.

## 5. Conclusions

This study indicates that the improvement of GCS was delayed compared to GRS and GLS in TTS patients. While LV systolic function, as assessed via LVEF, showed rapid recovery, GCS was observed to lag in complete recovery. CMR-FT may detect subtle impairment of LV function during the recovery process in patients with TTS.

## Figures and Tables

**Table 1:** Baseline characteristics.

Variable	All (*n* = 18)
Age	75.1 ± 10.4
Female	14 (77.8%)
Body mass index (kg/m^2^)	21.6 ± 3.2
Hypertension	9 (50.0%)
Diabetes	3 (16.7%)
Dyslipidemia	4 (22.2%)
COPD	2 (11.1%)
Asthma	1 (5.6%)
Malignancy	4 (22.2%)
Psychological disease	2 (11.1%)
Neurologic disease	1 (5.6%)
Current smoker	0 (0.0%)
Symptom at admission	
Chest pain	9 (50.0%)
Dyspnea	6 (33.3%)
Triggers	
Emotional stress	2 (11.1%)
Physical stress	9 (50.0%)
No apparent trigger	7 (38.9%)
ECG findings at presentation	
ST elevation	7 (38.9%)
QTc, msec	457.3 ± 45.7
Biochemical parameters on admission	
WBC, 10^3^/μL	8.9 ± 5.7
Creatinine, mg/dL	1.2 ± 1.3
CK, IU/L	233.6 ± 199.2
Troponin T, ng/mL (*n* = 14)	0.3 ± 0.4
Troponin I, pg/mL (*n* = 3)	6245.8 ± 10,108.4
BNP, pg/mL (*n* = 4)	678.4 ± 619.8
NT-pro BNP, pg/mL (*n* = 12)	7856.5 ± 16,807.3
LV ejection fraction (TTE), %	46.5 ± 17.1
Ballooning type	
Apical ballooning type	11 (61.1%)
Midventricular type	4 (22.2%)
Basal type	1 (5.6%)
Focal type	2 (11.1%)
Medication on admission	
ACE inhibitor or ARB	4 (22.2%)
Beta-blocker	3 (16.7%)
Antiplatelet	2 (11.1%)
Medication at discharge	
ACE inhibitor or ARB	8 (44.4%)
Beta-blocker	9 (50.0%)
Antiplatelet	2 (11.1%)

Values are shown as mean ± SD, or *n* (%). COPD = Chronic obstructive pulmonary disease; WBC = white blood cell; BNP = Brain natriuretic peptide; NT-pro BNP = N-Terminal pro Brain Natriuretic Peptide; ECG = electrocardiography; QTc = corrected QT interval; CK = creatine kinase; LV = left ventricular; TTE = transthoracic echocardiography. ACE inhibitor = angiotensin-converting-enzyme inhibitor; ARB = angiotensin-receptor blocker.

**Table 2:** Clinical events during hospitalization.

Variable	All (*n* = 18)
Heart failure	8 (44.4%)
Dyspnea on ventilator	3 (16.7%)
Cardiogenic shock	3 (16.7%)
Catecholamine use	3 (16.7%)
IABP	2 (11.1%)
LV thrombus	0 (0.0%)
Complete AV block	1 (5.6%)
Ventricular tachycardia	0 (0.0%)
Stroke	1 (5.6%)
All-cause death	0 (0.0%)

Values are shown as *n* (%). IABP = intra-aortic balloon pumping; LV = left ventricular; AV = atrioventricular.

**Table 3:** Serial findings on cardiovascular magnetic resonance.

Variable	1 Month	1 Year	*p* Value
LV ejection fraction, %	55.8 ± 9.2	58.9 ± 7.3	0.093
LVEDV, mL	81.5 ± 27.0	82.2 ± 22.7	0.91
LVESV, mL	36.4 ± 15.2	34.2 ± 12.9	0.54
LVSV, mL	45.1 ± 15.5	48.0 ± 12.8	0.41
LV mass, g	84.4 ± 27.1	80.9 ± 25.1	0.33
GCS (%)	−16.7 ± 3.4	−18.5 ± 3.2	<0.01
GRS (%)	59.6 ± 24.2	59.4 ± 17.3	0.95
GLS (%)	−12.8 ± 5.9	−13.8 ± 4.9	0.42

Values are shown as mean ± SD. LV = left ventricular; LVEDV = LV end-diastolic volume; LVESV = LV end-systolic volume; LVSV = LV stroke volume; GCS = global circumferential strain; GRS = global radial strain; GLS = global longitudinal strain.

## Data Availability

The data presented in this study are available on request from the corresponding author.

## Supplementary Materials

The following supporting information can be downloaded at: https://www.mdpi.com/article/10.3390/jcm13113238/s1, Table S1: Baseline characteristics of patients who were initially enrolled with a diagnosis of TTS; Table S2: Clinical events in hospitalization of patients who were initially enrolled with a diagnosis of TTS.
