# Supplementary material for: Time Course of Left Ventricular Strain Assessment via Cardiovascular Magnetic Resonance Myocardial Feature Tracking in Takotsubo Syndrome
Source: J Clin Med. 2024 May 30;13(11):3238. doi: 10.3390/jcm13113238 (PMC11172486; doi:10.3390/jcm13113238)
Supplement: Supplementary file 1 [file jcm-13-03238-s001.zip › Table S1 takotsubo MRI.pdf]

Table S1. Baseline characteristics

Variable	All (n=95)
Age	74.1±10.8
Female	79 (83.2%)
Body mass index (kg/m^2)	20.9±3.7
Hypertension	47 (49.5%)
Diabetes	14 (14.7%)
Dyslipidemia	26 (27.4%)
Current smoker	14 (14.7%)
Symptom at admission	
Chest pain	35 (36.8%)
Dyspnea	25 (26.3%)
Triggers	
Emotional stress	16 (16.8%)
Physical stress	60 (63.2%)
No apparent trigger	24 (25.3%)
ECG findings at presentation	
ST elevation	42(44.2%)
QTC, msec	464.6±56.3
Maximal CK myocardial band (U/L)	19.1±20.5
LV ejection fraction (TTE), %	50.7±12.6
Ballooning pattern	
Apical ballooning type	60 (63.2%)
Midventricular type	27 (28.4%)
Basal type	2 (2.1%)
Focal type	5 (5.2%)

Values are shown as mean ± SD, median(IQR), or n(%), CK = creatine kinase, ECG = electrocardiography; QTc = corrected QT interval; LV = left ventricular;
