# Supplementary material for: Time Course of Left Ventricular Strain Assessment via Cardiovascular Magnetic Resonance Myocardial Feature Tracking in Takotsubo Syndrome
Source: J Clin Med. 2024 May 30;13(11):3238. doi: 10.3390/jcm13113238 (PMC11172486; doi:10.3390/jcm13113238)
Supplement: Supplementary file 1 [file jcm-13-03238-s001.zip › Table S2 takotsubo MRI.pdf]

Table S2. Clinical events in hospitalization

Variable	All
In hospitalization	(n=95)
Heart failure	27 (28.4%)
Ventilator	11 (11.6%)
shock	17 (17.9%)
Catecholamine	16 (16.8%)
IABP	7 (7.4%)
LV thrombus	3 (3.2%)
Complete AV block	4 (4.2%)
Ventricular tachycardia	4 (4.2%)
Stroke	2 (2.1%)
All-cause death	7 (7.4%)
Cardiovascular death	1 (1.1%)
